# The amylase gene cluster in house mice (*Mus musculus*) was subject to repeated introgression including the rescue of a pseudogene

**DOI:** 10.1186/s12862-020-01624-5

**Published:** 2020-05-15

**Authors:** Miriam Linnenbrink, Kristian K. Ullrich, Ellen McConnell, Diethard Tautz

**Affiliations:** Max-Planck Institute for Evolutionary Biology, 24306 Plön, Germany

**Keywords:** Amylase gene cluster, Copy number variation, *Mus musculus*, Natural populations, Introgression

## Abstract

**Background:**

Amylase gene clusters have been implicated in adaptive copy number changes in response to the amount of starch in the diet of humans and mammals. However, this interpretation has been questioned for humans and for mammals there is a paucity of information from natural populations.

**Results:**

Using optical mapping and genome read information, we show here that the amylase cluster in natural house mouse populations is indeed copy-number variable for *Amy2b* paralogous gene copies (called *Amy2a1* - *Amy2a5*), but a direct connection to starch diet is not evident. However, we find that the amylase cluster was subject to introgression of haplotypes between *Mus musculus* sub-species. A very recent introgression can be traced in the Western European populations and this leads also to the rescue of an *Amy2b* pseudogene. Some populations and inbred lines derived from the Western house mouse (*Mus musculus domesticus*) harbor a copy of the pancreatic amylase (*Amy2b*) with a stop codon in the first exon, making it non-functional. But populations in France harbor a haplotype introgressed from the Eastern house mouse (*M. m. musculus*) with an intact reading frame. Detailed analysis of phylogenetic patterns along the amylase cluster suggest an additional history of previous introgressions.

**Conclusions:**

Our results show that the amylase gene cluster is a hotspot of introgression in the mouse genome, making it an evolutionary active region beyond the previously observed copy number changes.

## Background

The analysis of the evolution of the amylase locus in mammals has revealed different histories of duplication and specialization into salivary (*Amy1*) and pancreatic (*Amy2b*) amylases [1]. In the human lineage, gene copy number gains of *Amy1* led to increased expression of the AMY1 enzyme in human saliva, correlated to starch-rich diet shifts [2]. In dogs, copy number variation at the *Amy2b* gene has been linked to an increasing starch rich diet during domestication [3, 4] and analysis of amylase clusters across mammals has confirmed such a general tendency [1]. However, it has also been noted that diet correlation cannot fully explain copy number variation patterns in humans, especially since the AMY1 protein in the saliva has only a very limited role in starch digestion [5]. Physiological studies in humans have indeed yielded a more differentiated picture, including a possible role of amylase copy number in shaping the microbiome [6].

The house mouse (*Mus musculus*) forms a species complex with several described and not yet fully described sub-species [7–9] that are distributed in allopatric patterns across the whole world. Currently, three major lineages of *Mus musculus*, classified as subspecies, are distinguished: the Western house mouse *Mus musculus domesticus*, the Eastern house mouse *Mus musculus musculus* and the Southeast-Asian house mouse *Mus musculus castaneus*. All three lineages diverged roughly 0.5 million years ago in the area of the Iranian plateau [9]. During the past 10,000 years house mice have developed commensalism with humans, which allowed them to a spread across the world. The Western house mouse (*M. m. domesticus*) invaded Western Europe about 3000 years ago from a source population in Iran, via the Mediterranean route [9, 10]. From there it spread quickly across Western Europe, implying that the populations found in Western Europe have split not more than 3000 years ago. The Eastern house mouse (*M. m. musculus*) spread a few thousand years earlier from Asia into Eastern Europe [11] and it forms nowadays a contact zone with *M. m. domesticus* in the Middle o f Europe, where hybrids of the two subspecies can be found [12–14]. *M. m. castaneus* has mostly spread into Middle and Eastern Asia at unknown times, but presumably also as commensal with the spread of human agriculture [7, 8].

The sister species *Mus spretus* lives in sympatry with *M. m. domesticus* in Western Europe and can form partially sterile hybrids with this subspecies, but maintains its own species status.

Patterns of introgression between the Western and Eastern house mouse (*M. m. domesticus* and *M. m. musculus*) have revealed that introgression of haplotypes occurs not only at the hybrid zone in the middle of Europe, but also across large distances, possibly mediated through human mediated transport of mice [15]. A prominent example of a very recent introgression concerns a locus that confers resistance to the rodenticide Warfarin, *Vkorc1*, which has likely come from another mouse species related to *M. spretus* [16, 17]. But haplotypes may also introgress between sub-species or even between separated populations of the same subspecies, as it has been shown for the MLV virus receptor *Xpr1* [18].

In our previous genome-wide analysis of selective sweeps based on genome-wide microarray SNP data of two *M. m. domesticus* and two *M. m. musculus* wild populations, we found many mutually introgressed haplotypes between the house mouse subspecies, but mostly only at low frequency [15]. Still, simulation showed that these patterns can only be explained through adaptive mechanisms, especially for the long introgressed haplotypes. Among these long introgressed haplotypes, the chromosomal region including the amylase gene cluster stood out by showing fixed introgressed haplotypes in one of the populations, implying a rather strong recent selective sweep [15].

Here we study this region in much more detail, based on the genome sequencing data for the originally studied populations, as well as from four additional populations as represented in the data published by Harr et al. [19]. These include three *M. m. domesticus* populations, the one from Iran that is considered to be the source population for the mice that have arrived in Western Europe, as well as one from Southern France (Fra) and one from Western Germany (Ger). Further we include three *M. m. musculus* populations, one from Afghanistan (Afg) that is considered to be close to the source population of the animals that have spread into Asia and Eastern Europe, one from Kazakhstan (Kaz) and one from the Czech Republic (Cze), which is close to the hybrid zone (see Fig. 1 in [19] for a map). Finally, we include animals from one *M. m. castaneus* population, as well as from *M. spretus* as outgroup.

**Fig. 1:**
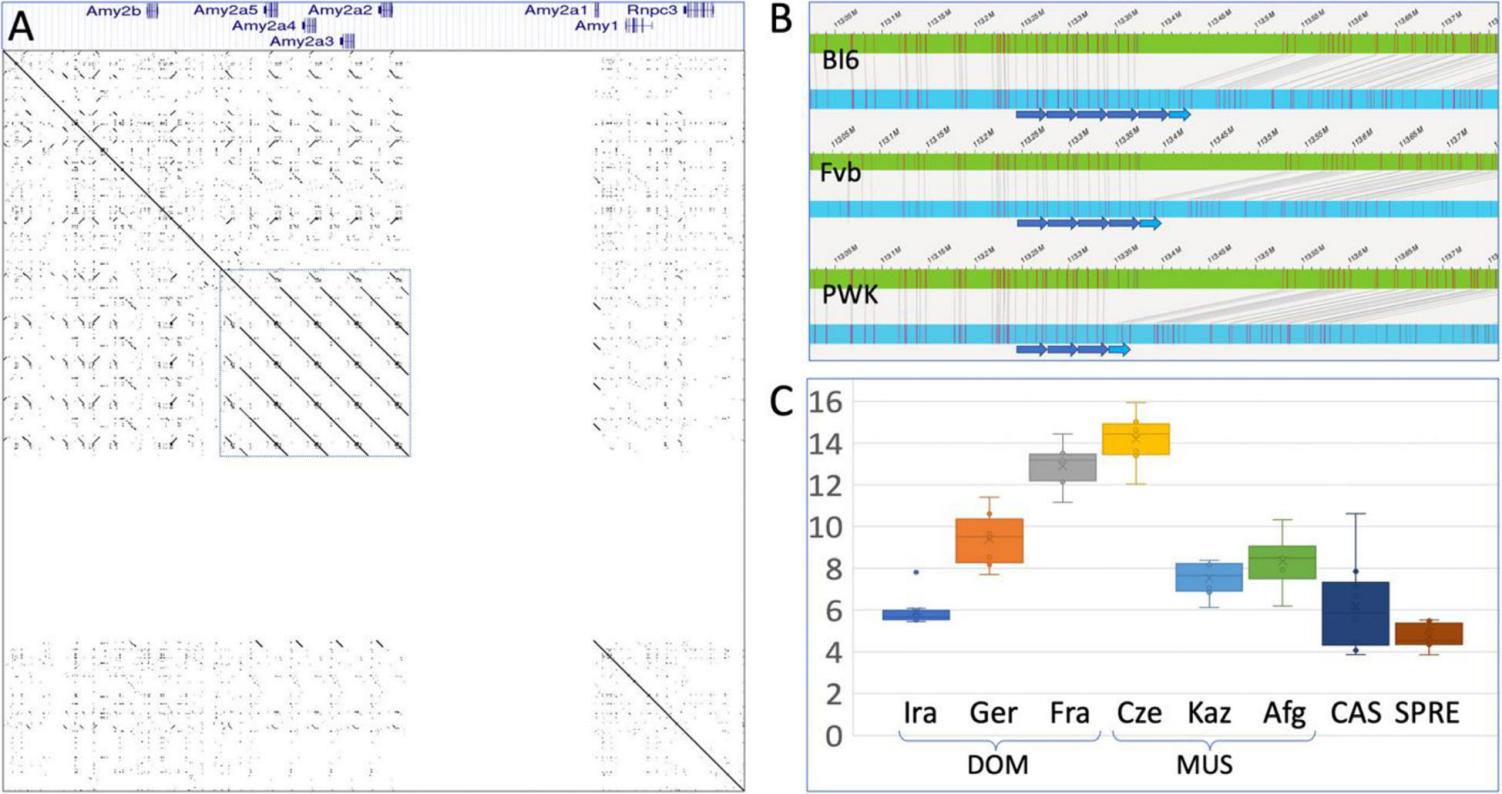
The amylase cluster genome region and structural variation. **a** Genomic organization and dotplot of the amylase gene cluster region in the mm10 reference genome (600 kb, representing positions chr3:113,050,000–113,650,000). The dot plot shows the internal repeats (boxed) and the annotation gap (visible as blank area). The gene annotations on the top are taken from the UCSC browser. It shows the sense strand, but the genes are encoded on the antisense strand. **b** Snapshots of the Bionano mapping results from the same genome region. The green bars represent the mm10 reference sequence, the blue bars the test genomes (C57Bl6, Fvb and PWK from top to bottom). **c***Amy2a* copy number ranges in the individuals of different wild populations, based on sequencing read coverage data in the region including the *Amy2a* repeats of the reference genome mm10 (150 kb, dotted square in (**a**)). Note that reads from the *Amy2b* region would also map to this, but since this is a single copy locus, it would not contribute to the variance caused by copy number changes

While the previous SNP dataset [15] was more limited and biased towards *M. m. domesticus* derived SNPs, the sequencing data allow the full resolution of the introgression patterns around the amylase gene region, only limited by the known problems of mapping short reads in copy-number variable regions. To address also the question of copy number variation that is of special importance for the amylase cluster, we use a combination of long-range optical mapping approaches (Bionano) with copy number estimation based on read depth.

We provide evidence that there is indeed copy number variation and introgression within and between populations. The most recent introgression correlates with a rescue of a pseudogenized *Amy2b* allele in populations in France. However, we noticed also that the introgression region as a whole has a much more complex history, with apparent multiple introgression events in different directions and from different outgroups, leading to complex phylogenetic topologies throughout the region. Hence, the evolutionary dynamics at the amylase locus goes beyond the effects of copy number variations.

## Results

### The amylase cluster genomic region

In the mm10 reference sequence, the amylase cluster is encoded on the antisense strand. Figure 1a depicts its organization on the canonical sense strand, i.e. the order is reverse to the numbering of the individual genes in the cluster. It includes from 5’to 3’ the *Amy2b* gene, tandem repeats with four full and one partial *Amy2a* paralogous genes (named *Amy2a5* - *Amy2a1*), an annotation gap of 150 kb and a single *Amy1* gene (Fig. 1a). The gap lies within the *Amy2a1* repeat region, i.e. the annotators of the reference genome suspected at least one extra copy.

We have used the Bionano long-range optical mapping technology to resolve this region in three inbred strains from *M. m. domesticus* and *M. m. musculus* and one *M. m. musculus* wildtype individual (Fig. 1b, Suppl. Fig. 1). We find that C57Bl6, which is the source for the mm10 mouse (*M. m. domesticus*) reference genome, has one additional copy compared to the reference sequence, i.e. the annotated gap could be removed by adding one more *Amy2a* repeat into the reference sequence. The inbred strain Fvb (also derived from *M. m. domesticus*) shows four repeat units, the inbred strain PWK (derived from *M. m. musculus*) has three copies (Fig. 1b). When running a haplotype aware de novo assembly for the Bionano data, we still recover only one length allele for each inbred strain, but two length alleles for the outbred *M. m. musculus* individual from the Kaz population (Suppl. Fig. 1). These observations suggest that copy number at the amylase locus is not hypervariable, i.e. there is no variation within each of the inbred strains. But a wild derived animal can carry two haplotypes, indicating polymorphism within wild populations.

To more systematically assess copy number ranges in natural populations, we used genome read counts mapped to the annotated repeat region as a measure for copy number variation in the individuals (*N* = 6–10, see Methods) for each of the different populations (*N* = 8) described in [19] (Fig. 1c). We find some variation within populations (especially in *M. m. castaneus*), but also differences in the average between populations. Diploid copy numbers of *Amy2a* paralogs range between 4 and 16, with the highest numbers found in the *M. m. domesticus* population from Fra and the *M. m. musculus* population from Cze. House mice are omnivorous [20], i.e. use both plant material (e.g. seeds), as well as animal material (e.g. worms, insects) as diet. Neither of the subspecies or populations has been reported to be specialized on starch-rich food and the mandible shapes correspond to typical omnivorous rodents [21]. However, given that most were caught in the vicinity of agricultural storage places, it would seem likely that their food is biased towards usage of grains. Analysis of feral mice coming from non-commensal regions has shown that their mandible shapes change towards a more carnivorous type, possibly due to plasticity effects [21–24], implying that the commensal life-style is characterized by more starch-rich diets. Given this general observation and the fact that the mice studied here have a common commensal origin, one would have expected similar *Amy2a* copy numbers, if these are a reflection of adaptation to starch-rich diet. However, we find major copy number differences, especially between the Fra and Ger populations that were caught in very similar habitats. This allows to conclude that there is no direct support for an adaptive correlation of *Amy2a* copy numbers to starch-rich diet in mice.

### Genomic sequence data

**Fig. 2:**
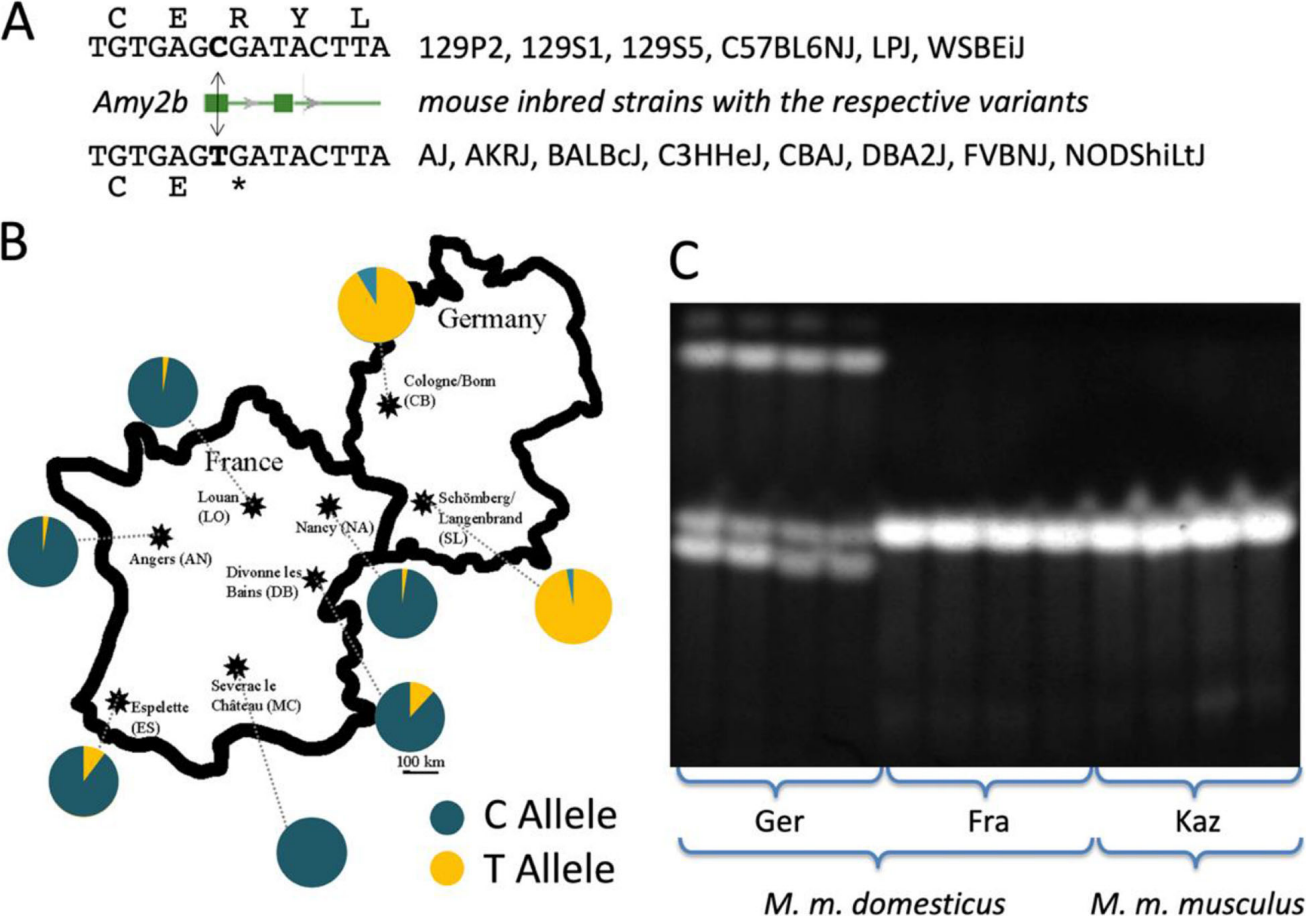
*Amy2b* haplotypes and distribution patterns. **a** Depiction of the disabling mutation in exon 1 of *Amy2b* and list of inbred mouse strains that carry either of the variants. The sequence shown corresponds to positions chr3:113,156,570-113,156,584 in the mm10 genome reference sequence. **b** Frequencies and distribution of the disabling (T-allele) versus enabling (C-allele) in additional different *M. m. domesticus* populations in France and Germany taken from [25]. **c** Native gel of pancreas samples stained with amylase activity assay for four animals each of the indicated populations. The Ger population displays an array of three different variants, each with lower activity. Note that a denaturing gel would not resolve these variants (data not shown). The Fra and Kaz animals show only one variant with strong activity

Inspection of the amylase sequences from the genomic re-sequencing data [19] provides a possible explanation for the adaptive introgression of a *M. m. musculus* haplotype including the *Amy2b* gene into Fra that we had found based on microarray SNP data in [15]. All of the Ger individuals sequenced harbor a mutation in the first exon that leads to a premature stop codon (Fig. 2a). Hence, the sequenced Ger individuals carry a pseudogene for *Amy2b*. In fact, 8 of the 14 fully sequenced mouse inbred strains derived from *M. m. domesticus* harbor the same stop codon (Fig. 2a).

Based on a PCR assay, we typed this variant for an extended sample of animals and populations and found the pseudogene to be prevalent in populations from Germany, but rare in populations from France (Fig. 2b). A native gel electrophoresis from pancreas tissue shows that there are also active amylase variants in the Ger individuals, but the band pattern differs clearly from the one found in the Fra animals. While the Ger animals show three bands, the Fra animals show only one, which is the same as the *M. m. musculus* animals from Kazakhstan (Fig. 2c). Given the pseudogene status of *Amy2b* in Ger, the three bands are likely derived from the *Amy2a* paralogous copies, while the strong band in Fra and Kaz would likely represent the *Amy2b* single copy gene. Interestingly, Fra and Kaz do not show additional bands, although they have paralogous *Amy2b* copies as well (Fig. 1c). This could suggest that in these haplotypes, the paralogues are actually not expressed, implying that there are also regulatory effects (i.e. silencing of paralogues) associated with this haplotype. However, detailed chromatin and proteomic analysis would be required to proof this conjecture. In any case, the gel shows that the haplotypes are very different between Ger and Fra and that this could be a basis for differential adaptive effects.

### Complex introgression patterns

**Fig. 3:**
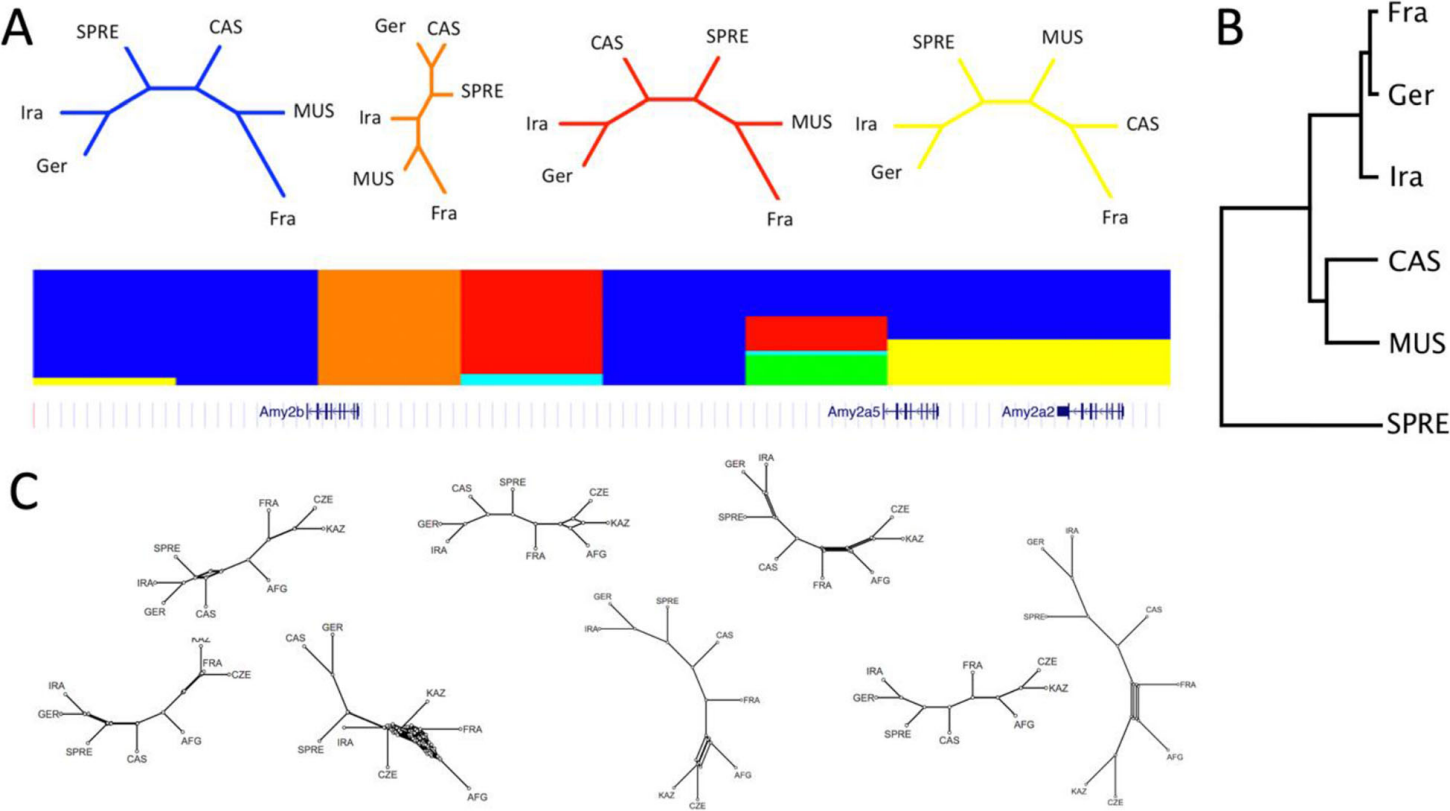
Comparison of tree topology changes in the region around *Amy2b* based on *Twisst* and *PoMo*. **a** Eight consecutive windows of 25 kb are depicted as colored blocks. The colors match with the trees shown on the top (the green and light blue trees are not shown). Different blocks in a given window represent the different probabilities for the respective trees. The relative location of *Amy2b* and two of its paralogs are shown at the bottom. **b** Expected tree topology for the five taxa [9] (compare also to the tree in Fig. 5 from a random genome region). Taxa designations as in Fig. 1, in addition SPRE represents *M. spretus*. **c***PoMo* split trees for the same 25kbp windows as in **a**

To further characterize the introgression pattern around the *Amy2b* region, we applied the *Twisst* algorithm for visualizing phylogenetic incongruence through topology weighting in large datasets [26]. This algorithm is designed to resolve more details in regions where introgression events had been detected. It finds the best supported topologies for a given genomic window in a dataset with multiple individuals. Next to *Twisst* we applied polymorphism-aware phylogenetic models (*PoMo*) using site-frequency data with *IQ-TREE* [27]. *PoMo* builds on top of DNA substitution models and accounts for incomplete lineage sorting. For *Twisst* we used the sequences of all individuals from six of the populations (48 individuals in total) and constrained the output to a 6-taxon topology, since the full topologies would be too complex. Figure 3a displays the results for the 200 kb region around *Amy2b* in consecutive 25 kb windows. It starts with two windows (depicted in blue) where Fra and MUS are sister groups and SPRE lies between [Ira,Ger] and CAS. In the third window (orange) CAS is a sister group of Ger to the exclusion of Ira - note that this is the window that includes exon1 of *Amy2b*, since the gene is coded on the antisense strand. In the fourth window (red), Ira and Ger are again sister groups, while SPRE is between CAS and MUS. The further windows represent mostly the blue tree, with one additional major alternative tree (yellow) where CAS is sister group of Fra. None of these trees represents the expected species / sub-species tree where the *M. m. domesticus* populations (Ger, Fra, Ira) should group together and SPRE should be the outgroup (Fig. 3b). Hence, this analysis suggests that the actual introgression history is not just a single event, but is apparently much more complex. These findings are supported by the *PoMo* analysis, where additionally MUS was divided into three sub-populations (MUS-Afg, MUS-Cze, MUS-Kaz; Fig. 3c).

**Fig. 4:**
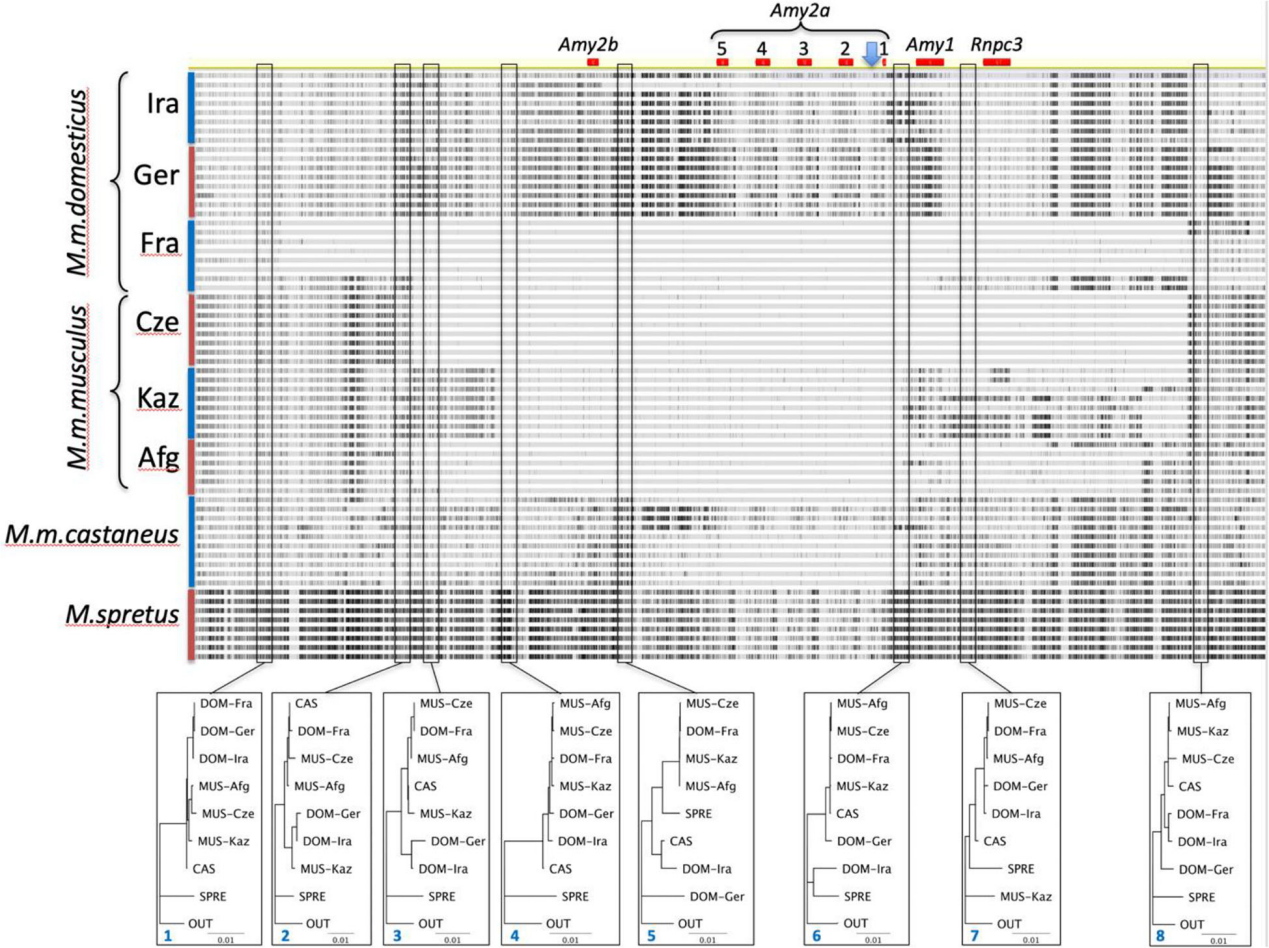
Alignments of individual sequences in the amylase region. The alignment is shown for the extended region (chr3: 112,700,001–114,300,000), the annotation of the amylase copies plus the neighboring gene (*Rnpc*3) is provided on the top. The annotation gap was removed, its position is marked by the blue arrow head. The sequences were aligned to the mm10 reference sequence, differences are indicated by black marks. The trees on the bottom represent bootstrap consensus trees of 10 kb sections from the positions indicated by red boxes. All nodes have 100% bootstrap support. The outgroup was *M. caroli*, but the branch length for the outgroup was trimmed

While the *Twisst* and *IQ-TREE* analysis are good approaches to detect anomalies, they are limited by applying a window approach and the topologies to be considered rise with the number of sequences/populations included. Hence, we used also a direct alignment visualization approach to get an impression on the complexity of evolutionary patterns in the extended amylase region. For this we used all individuals from all populations in the analysis and aligned them to the mm10 reference sequence. Difference to the reference sequence are highlighted as black bars for each sequence (Fig. 4).

It is evident that different populations harbor different haplotype blocks, at least partially. Since the reference sequence reflects mostly the sequence that can be found in most of the individuals from Fra, these show the smallest number of substitutions throughout the region. We had previously shown that the haplotypes found in the Fra population are derived from *M. m. musculus* [15], and this is also visible in the alignment pattern. Interestingly, however, overlaps are different for the different *M. m. musculus* populations, but all include the *Amy2b* and *Amy2a* gene regions.

The visualization of alignment patterns generates necessarily a biased view, since the patterns depend on the reference sequence chosen. But one can use these patterns to analyze subregions of interest in phylogenic trees that represent all relationships in reference to an outgroup (here *Mus caroli*). We have done this for eight such subregions, covering 10 kb each. The respective trees are displayed at the bottom of Fig. 4. Regions 1 and 8 represent essentially the expected relationships, thus bounding the whole region. Actually, going further out of the region would mostly generate the same trees. But inside the region, basically every tree shows a different pattern, with mixed relationships between the taxa. This includes also trees where *M. spretus* is placed among the *M. musculus* populations (windows 5 and 7) or where a *M. m. domesticus* population (Ger) is placed as a sistergroup to *M. spretus* (window 6).

### Aminoacid substitution patterns

**Fig. 5:**
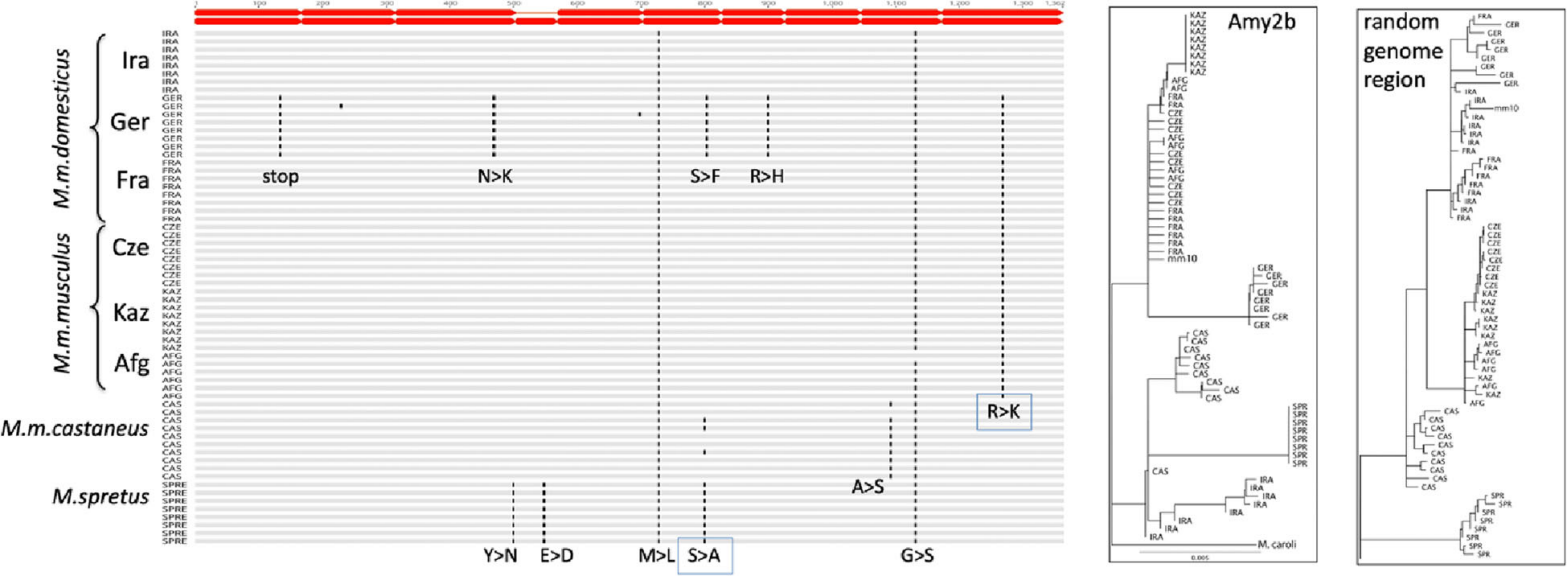
*Amy2b* protein sequence alignments between individuals of the different mouse populations. Substitutions are marked in reference to the outgroup sequence from *M. caroli*. The red block arrows at the top indicate the exons - note that one of the exons is alternatively spliced, i.e. two major protein variants are expected to exist. The *Amy2b* tree to the right is derived from this alignment, but includes also the information from the non-coding substitutions. The tree at the far right represents a randomly chosen genome region from chromosome 3 (chr3:54,483,946–54,586,356) which reflects the expected topologies among the subspecies

To explore whether these apparent repeated events of introgression could reflect cycles of selection on different protein variants, we have analyzed the substitution patterns in the *Amy2b* gene among the individuals of the different populations (Fig. 5). The Ger population harbors a total of four population-specific substitutions, including the stop codon described above. Given the origin of the Western European populations (Ger and Fra) from Iran, one would have expected that at least the Ira population would harbor some of them, especially since it has maintained a large effective population size over time (suppl. Fig. 2). Given that this is not the case, it would seem the Ger *Amy2b* haplotypes are derived from an unknown distant source, which is also clear from the long branch in the corresponding tree (Fig. 5). Note that this tree does generally not reflect the expected topology that can be obtained from a randomly chosen region outside of the amylase cluster (Fig. 5). In the *Amy2b* tree, the Fra sequences group within the *M. m. musculus* populations and neither the IRA nor the SPR (*M. spretus*) sequences follow the expected topologies. Note that the SPR sequences of all eight individuals are also almost identical to each other, although the population as a whole is highly polymorphic and has maintained a high N_e_ over time (suppl. Fig. 2), suggesting that they may have also been subject to a separate recent sweep event. Hence, the overall pattern of the protein alignment suggests also a complex history of events which do not yield further clues about possible causative substitutions, apart of the stop codon mutation in Ger.

## Discussion

Similar as in other mammals [1], we find copy number variation of amylase genes between species and sub-species of house mice. However, a direct connection to starch diet differences is not evident. The two Western European populations studied (Ger and Fra) show major differences in copy number, but both live commensal in similar agricultural contexts. On the other hand, the copy number difference between the Western European *M. m. domesticus* populations can at least in part be explained by an introgression of a *M. m. musculus* haplotype into the Fra population. Intriguingly, by studying the details of this introgression in the context of multiple populations and outgroups, we found patterns that are more compatible with a history of multiple introgressions from different sources and in different combinations among these sub-species and species. This implies that statistical procedures that test single scenarios come to their limits, especially when they are based on the comparison of site frequency spectra. But visualizing a multiple alignment pattern as we have done it in Fig. 4 can identify the haplotype blocks and breakpoints rather well, albeit only visually.

The most recent introgression event appears to be the one that has led to the major difference between the Fra and the Ger populations. The eight sequenced individuals of each population show completely different haplotypes, whereby the Fra haplotypes are identical to each other as well as the *M. m. musculus* CZE animals (suppl. Fig. 3). This implies that the Cze population has undergone a similar recent sweep, but since we have less phylogeographic information from the neighboring populations in this case, a more detailed scenario is not possible. For the Western European populations, however, the larger population sample depicted in Fig. 2b indicates that the mutual other haplotype can be found in low frequencies in each of the populations.

One can therefore propose a scenario in which the *M. m. domesticus* populations invading Western Europe about 3000 years ago [10] have acquired a haplotype with a stop codon in the first exon of the *Amy2b* gene from an unknown source. While this should have impacted the pancreas amylase production, it would not have abolished it completely, since the *Amy2a* duplicated copies still appear to produce some functional protein (compare Fig. 2c) and their copy number variation might at least partially compensate for the loss of the *Amy2b* reading frame. Hence, although possibly maladaptive to some degree, the stop codon could have become fixed (or almost fixed) through drift during the colonization phase. A second introgression from a *M. m. musculus* animal would have occurred in the Fra area relatively recently and this has led to a fast (i.e. < 3000 years) replacement of the haplotype including the *Amy2b* pseudogene. Parts of this haplotype including the whole amylase gene cluster are seen also in all three *M. m. musculus* populations (section between regions 4–6 in Fig. 4 and suppl. Fig. 3 with the genomic alignments across the *Amy2b* and *Amy1* genes), but each of these populations differs with respect to flanking sequences, supporting also the notion of a complex introgression history among these populations.

Incomplete lineage sorting (ILS) would be an alternative to introgression, although we had already previously shown that this is unlikely to explain most of the introgression patterns seen in wild mouse populations [15]. However, there are a number of further reasons why ILS can practically be excluded in the present case. The introgression signals are highly localized around the amylase cluster, nothing similar can be seen in its chromosomal vicinity as is evident from analyzing the F_st_ patterns in the region (suppl. Fig. 4). Even across the genome, it is among the strongest introgression signals [15]. ILS, on the other hand, should be more or less evenly distributed across the genome, since it is a consequence of random drift. Indeed, ILS patterns are typically seen in comparison among individuals from the subspecies (e.g. the tree from the random genome region shown in Fig. 5). But the sub-species are expected to have split about 0.5 Mya and sharing of haplotypes cannot be explained by ILS alone [28]. Hence, while it is very difficult to distinguish between ILS and introgression in any given region of the genome, the overall pattern within the amylase cluster is much more compatible with repeated events of introgression between sub-species.

## Conclusion

Our data suggest that the evolutionary dynamics around the amylase cluster in mice include repeated presumably adaptive introgression events and thus go beyond copy number variation and adaptation to starch rich diets. Whether this is also the case for other species is as yet open. In humans, the respective amylase gene repeats are too similar to each other to allow unequivocal short read mapping, i.e. a deeper analysis will have to await long read datasets from population samples. In dogs, Reiter et al. [4] have analyzed whether introgression from wolves may have occurred into dog breeds. They found no clear evidence for this, but the analysis was much shallower than the one we have done here. Reiter et al. found also that the correlation between copy number and starch diet is more complex in dogs and suggest that additional factors may be relevant, similar as it has been suggested in humans [5, 6]. We note that although the by far highest expression of amylases is in the salivary glands and the pancreas in mice, there is also reasonably high expression in the gut, the spleen and the thyroid and the expression in the gut is even different between the different populations (suppl. Fig. 4). Hence, there may be additional unnoticed functions of the amylase genes that could contribute to the observed evolutionary dynamics.

## Methods

### Ethics statement

The work did not involve in vivo experiments with animals. Mouse samples were taken from dead mice derived from the maintenance of the mouse strain collections at our institute. As part of the strain maintenance, surplus mice are killed by CO_2_ asphyxiation and organs were taken from such mice. Population DNA samples were taken from previous studies described in [19, 25]. Maintenance and handling of mice in the facility were conducted in accordance with German animal welfare law (Tierschutzgesetz) and FELASA guidelines. Permits for keeping mice were obtained from the local veterinary office ‘Veterinäramt Kreis Plön’ (permit number: 1401–144/PLÖ-004697).

### Data and analysis

Genome sequence data used in this study were taken from [19]. These include individuals representing natural populations of *M. m. domesticus* (24 individuals from 3 populations; 8 from Germany (Ger), 8 from France (Fra) and 8 from Ahvaz, Iran (Ira)), *M. m. musculus* (22 individuals from 3 populations: 8 from the Czech Republic (Cze), 8 from Kazhakstan (Kaz) and 6 from Afghanistan (Afg)), *M. m. castaneus* (10 individuals from one population in India (CAS)) and *M. spretus* (8 individuals from one population in Spain (SPRE)). Read coverage data for the amylase region were obtained with samtools (Li, et al. 2009) and calculated as the ratio of reads falling within the amylase region as compared to total mapped reads (*samtools view -c -F 4*). Conversion into copy numbers was done by applying a single factor calibrated on an average of 8 copies for the Kaz individuals (the optical mapping results for one such individual reveal two haplotypes, one carrying 4, the other three copies - see suppl. Fig. 1, i.e. 8 copies for the diploid seems a reasonable estimate). This calibration results in an average of 13 copies for the Fra individuals. Given that the Fra haplotype corresponds to the Bl6 haplotype (see Fig. 4) and optical mapping for Bl6 revealed 12 copies, we consider this calibration as reasonably consistent. Individual genomic sequences were retrieved by using *ANGSD* (Korneliussen et al. 2014) and the ‘doFasta 4’ option (*angsd -doFasta 4 -doCounts 1 -minQ 20 -minMapQ 0 -setMinDepth 5 -iupacRatio 0.25*). For the *Twisst* analysis phylogenetic trees per 25kbp windows were calculated with the ‘bionjs’ function of the R package ‘ape’ [29] and used as input files for *Twisst* [26]. For the *PoMo* analysis individual sequences were converted and grouped into population count data with *cflib* (https://github.com/pomo-dev/cflib) and phylogenetic trees were inferred per 25kbp windows with *IQ-TREE* (*iqtree -m HKY + P -bb 1000*). Data visualization was done based on the UCSC genome browser for the mouse assembly mm10 and the Geneious Prime software (v 2019.03). The dotplot was generated with Gepard 1.4 [30]. The *PoMo* trees were visualized as split networks with SplitsTree5 (Huson and Bryant 2006). The SMC++ network was generated as described in [31] using the extended sequence information in the present paper.

### Mouse population analysis

DNA of mouse samples described in [25] was used to amplify the fragment covering the predicted stop codon. The resulting PCR fragments were sequenced by Sanger sequencing and the sequences were manually inspected for the presence of the C/T polymorphism.

### Bionano optical mapping

Optical mapping was done for three inbred mouse strains (C57Bl6, PWK and FVB) and one wild-derived outbred mouse strain (MUS), following the standard procedures on a Bionano Saphyr instrument using Nt.BspQI, which labels GCTCTTC strings (Nt.BspQI) in the DNA. Extraction of megabase genomic DNA was done according to the Saphyr Bionano Prep Animal Tissue DNA Isolation Soft Tissue Protocol (Document Number: 30077; Document Revision: B). Briefly, cell nuclei were isolated from spleen tissue and embedded in agarose plugs. High molecular weight (HMW) DNA was purified with proteinase K and RNAse in plugs, genomic HMW DNA was further extracted from agarose plugs and cleaned by drop dialysis. HMW DNA was homogenized overnight and quantified with the Qubit BR dsDNA assay and kept at 4 °C until labelling. The purified HMW DNA was labelled according to the Bionano Nick Label Repair and Stain (NLRS) protocol (Document Number: 30206 Revision: C; Document Number: 30024 Revision: I). For NLRS, the enzyme Nt.BspQ1 was used to nick 900 ng HMW DNA. After the clean-up step, the HMW DNA was pre-stained, homogenized, and quantified with the Qubit HS dsDNA assay to use an appropriate amount of backbone stain YOYO-1. The molecules were imaged using the Bionano Saphyr system (Bionano Genomics, San Diego).

### Bionano de-novo assemblies

The optical mapping de-novo assemblies were performed using Bionano Solve (Solve3.5.1_01142020) with RefAligner (10,330.10436rel) using the default Bionano parameter file for a non-haplotype aware assembly without the ‘extend and split’ option with the ‘Cut CMPR’ option and with a pre-assembly to automatically set the noise parameters (Nt-BspQI: optArguments_nonhaplotype_noES_saphyr.xml) using mm10 (ftp://ftp.ensembl.org/pub/current_fasta/mus_musculus/dna/Mus_musculus.GRCm38.dna.primary_assembly.fa.gz) as the reference. For a haplotype-aware local re-assembly of the amylase region the command-line version of Bionano Solve was used with the default Bionano parameter file for an EnFocus FSHD Analysis (Nt.BspQI: optArguments_haplotype_saphyr_human_D4Z4_guided.xml) using the amylase region as the seed (chr3:112,781,434-113,781,433). De novo assemblies were visualized with Bionano Access (1.5.1).

### Amylase purification and quantification

Approximately equal weights of pancreas per sample were used for purification. Tissues were homogenized in PBS using a TissueLyser II (Qiagen), centrifuged at 13,000 x g at 4 °C for 10 min, and the crude lysate was collected. Ethanol was added to a final concentration of 40%, centrifuged at 10,000 x g for 10 min at 4 °C, and the supernatant was collected. Amylase was precipitated by addition of 1 mg of oyster glycogen (Sigma-Aldrich) according to [32] followed by shaking on ice for 5 min. It was then pelleted by centrifugation at 5000 g for 3 min at 4 °C. The samples were washed, re-suspended in PBS, and glycogen digested by incubation at 30 °C for 20 min [33]. Samples were stored at − 80 °C in aliquots to avoid repeated freeze/thaw cycles. Protein concentration was determined using Thermo Scientific’s Coomassie Plus™ (Bradford) Assay kit according to the manufacturer’s instructions. For native PAGE gels Amylase extracts were separated on 7.5% Mini-PROTEAN® TGX™ gels (Bio-Rad), but in their native form (no boiling, no SDS). The gel was then placed in 1% pre-warmed starch solution and incubated for 1 h at 37 °C (without shaking). Subsequently it was transferred to Lugols solution for approx. 1 min and then straight to PBS. This was followed by two further brief washes in PBS and imaging using a white light transilluminator.

## Supplementary information

**Additional file 1: Supplementary Figure 1:** Local Haplotype-aware de-novo assembly using optical-mapping data around the amylase cluster on chromosome 3. The green bars represent the mm10 reference sequence, the blue bars the test genomes whereby always two inferred haplotype reconstructions are shown. For the inbred strains (Bl6, FvB and PWK) these are identical, for MUS (a *M. m. musculus* individual from the Kaz population), we find one haplotype with 4 copies and one with 3 copies. **Supplementary Figure 2:** Inferred population history for natural populations of the house mouse. SNP data from [19] was filtered to only retain intergenic regions without any feature annotation. For each population a separate smc++ [34] model was created setting the per generation mutation rate to 5 × 10^− 9^. **Supplementary Figure 3:***Amy1* and *Amy2b* full gene sequence alignments between individuals of the different mouse populations. Substitutions are marked in reference to the reference sequence from mm10. The yellow arrows at the top indicate the exons - note that one of the exons is alternatively spliced in *Amy2b*, i.e. two major protein variants are expected to exist. **Supplementary Figure 4:** Screenshot from UCSC browser tracks around the amylase cluster region on chromosome 3. Data in the tracks are taken from Harr et al. (2016) and accessibility to the tracks is described therein. The screen view here shows only a subset of the tracks. The top three tracks (green) are Fst measures in 10 kb windows and pairwise comparisons between the populations indicated to the left. The track scale was adjusted to display Fst > 0.5 only. It is evident that the amylase cluster stands out in all three comparisons between the three *M. m. domesticus* populations, where the most recent introgression events have occurred. The lower tracks represent the expression data, whereby the track scale was set to 0–10,000, implying that only highly expressed genes become visible. The mapping stringency for the expression data had a long stringency, i.e. the reads covering the *Amy2* genes could come from any of the loci, since due to their high similarity they would have been equally distributed between the loci. Note that the expression of the amylase genes in the gut is only seen for the IRA population. The bottom track displays the UCSC annotated genes in squish mode.

## Data Availability

The population genome sequencing data analyzed in this paper were all previously published in [19] which lists also all accession numbers to the depositions in the European Nucleotide Archive. The optical mapping data are available at: http://wwwuser.gwdg.de/~evolbio/evolgen/others/amylase/data/bionano/.

## Abbreviations

ILS: Incomplete lineage sorting
Mya: million years ago
M. m.: *Mus musculus*
Ger: Wild mouse population from Germany (area Cologne/Bonn)
Fra: Wild mouse population from France (area Massif Central)
Ira: Wild mouse population from Iran (area Ahvaz)
Cze: Wild mouse population from Czech Republic (area Studenec)
Kaz: Wild mouse population from Kazakhstan (area Massif Almaty)
CAS: *M. m. castaneus* wild mouse population from India (area Himachal Pradesh)
SPRE: *M. spretus* wild mouse population from Spain (area Madrid)
MUS: *M. m. musculus*
DOM: *M. m. domesticus*
SPR: *M. spretus*
