# Supplementary material for: The amylase gene cluster in house mice (Mus musculus) was subject to repeated introgression including the rescue of a pseudogene
Source: BMC Evol Biol. 2020 May 15;20:56. doi: 10.1186/s12862-020-01624-5 (PMC7227347; doi:10.1186/s12862-020-01624-5)
Supplement: Supplementary file 1 — Additional file 1: Supplementary Figure 1: Local Haplotype-aware de-novo assembly using optical-mapping data around the amylase cluster on chromosome 3. The green bars represent the mm10 reference sequence, the blue bars the test genomes whereby always two inferred haplotype reconstructions are shown. For the inbred strains (Bl6, FvB and PWK) these are identical, for MUS (a M. m. musculus individual from the Kaz population), we find one haplotype with 4 copies and one with 3 copies. Supplementary Figure 2: Inferred population history for natural populations of the house mouse. SNP data from [19] was filtered to only retain intergenic regions without any feature annotation. For each population a separate smc++ [34] model was created setting the per generation mutation rate to 5 × 10− 9. Supplementary Figure 3:Amy1 and Amy2b full gene sequence alignments between individuals of the different mouse populations. Substitutions are marked in reference to the reference sequence from mm10. The yellow arrows at the top indicate the exons - note that one of the exons is alternatively spliced in Amy2b, i.e. two major protein variants are expected to exist. Supplementary Figure 4: Screenshot from UCSC browser tracks around the amylase cluster region on chromosome 3. Data in the tracks are taken from Harr et al. (2016) and accessibility to the tracks is described therein. The screen view here shows only a subset of the tracks. The top three tracks (green) are Fst measures in 10 kb windows and pairwise comparisons between the populations indicated to the left. The track scale was adjusted to display Fst > 0.5 only. It is evident that the amylase cluster stands out in all three comparisons between the three M. m. domesticus populations, where the most recent introgression events have occurred. The lower tracks represent the expression data, whereby the track scale was set to 0–10,000, implying that only highly expressed genes become visible. The mapping stringency for the expressio [file 12862_2020_1624_MOESM1_ESM.docx]

**Supplementary Figures**

**
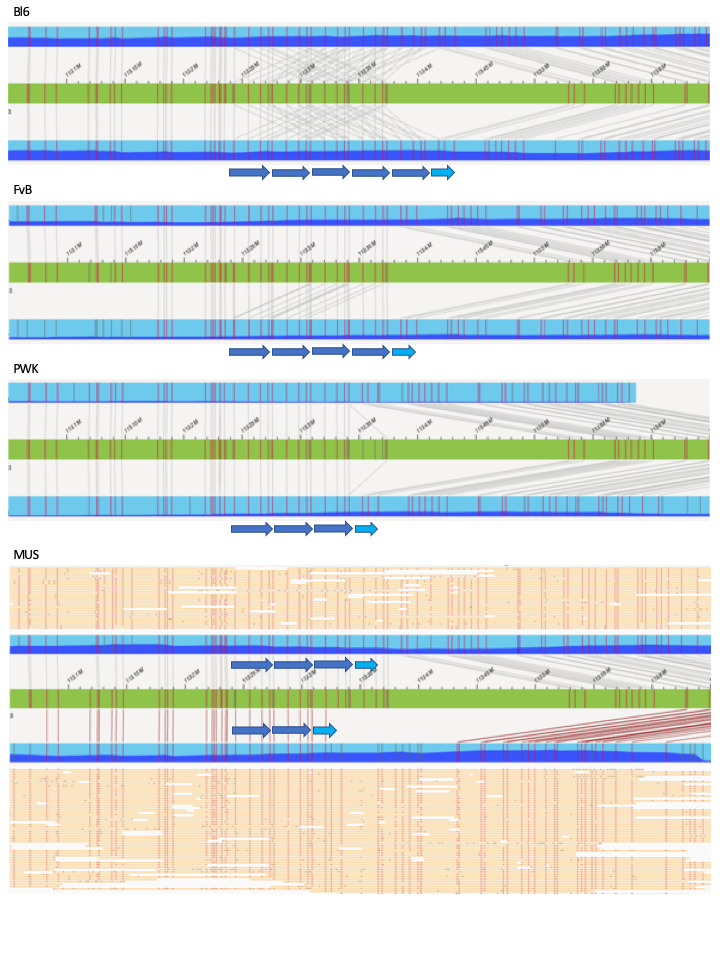
**

**Supplementary Figure 1:** Local Haplotype-aware de-novo assembly using optical-mapping data around the amylase cluster on chromosome 3. The green bars represent the mm10 reference sequence, the blue bars the test genomes whereby always two inferred haplotype reconstructions are shown. For the inbred strains (Bl6, FvB and PWK) these are identical, for MUS (a *M. m. musculus* individual from the Kaz population), we find one haplotype with 4 copies and one with 3 copies.


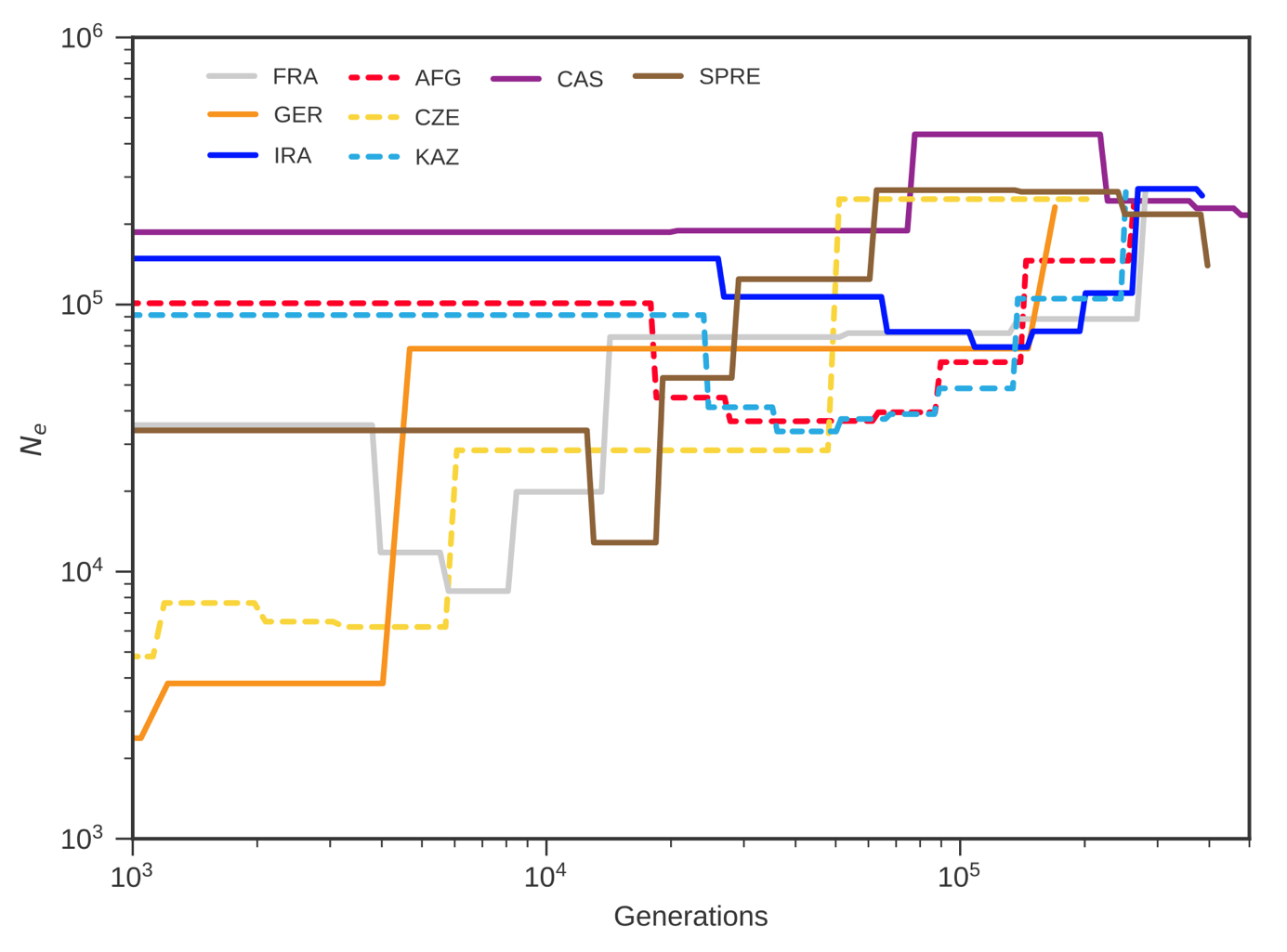


**Supplementary Figure 2:** Inferred population history for natural populations of the house mouse. SNP data from [19] was filtered to only retain intergenic regions without any feature annotation. For each population a separate smc++ [34] model was created setting the per generation mutation rate to 5 x 10^-9^.


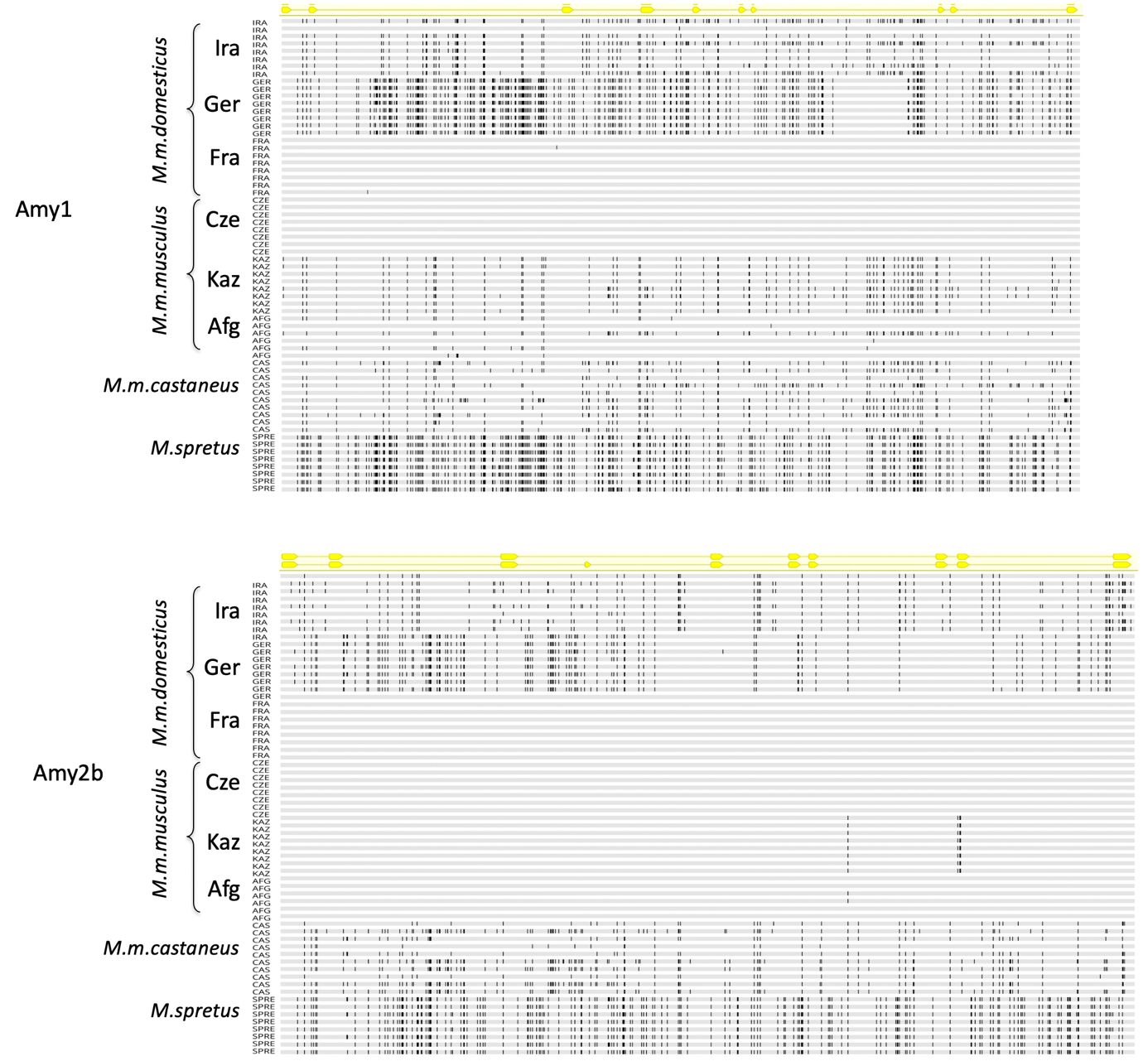


**Supplementary Figure 3:** *Amy1* and *Amy2b* full gene sequence alignments between individuals of the different mouse populations. Substitutions are marked in reference to the reference sequence from mm10. The yellow arrows at the top indicate the exons - note that one of the exons is alternatively spliced in *Amy2b*, i.e. two major protein variants are expected to exist.


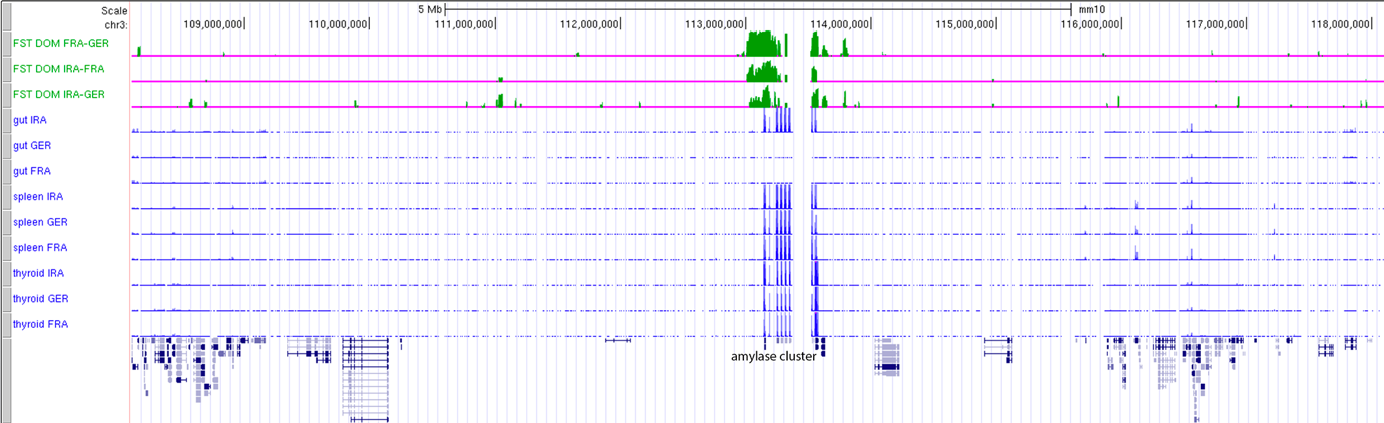


**Supplementary Figure 4:** Screenshot from UCSC browser tracks around the amylase cluster region on chromosome 3. Data in the tracks are taken from Harr et al. (2016) and accessibility to the tracks is described therein. The screen view here shows only a subset of the tracks.

The top three tracks (green) are Fst measures in 10kb windows and pairwise comparisons between the populations indicated to the left. The track scale was adjusted to display Fst > 0.5 only. It is evident that the amylase cluster stands out in all three comparisons between the three *M. m. domesticus* populations, where the most recent introgression events have occurred.

The lower tracks represent the expression data, whereby the track scale was set to 0-10,000, implying that only highly expressed genes become visible. The mapping stringency for the expression data had a long stringency, i.e. the reads covering the *Amy2* genes could come from any of the loci, since due to their high similarity they would have been equally distributed between the loci. Note that the expression of the amylase genes in the gut is only seen for the IRA population.

The bottom track displays the UCSC annotated genes in squish mode.
